# Supplementary material for: Viral Infection Induces Alzheimer’s Disease-Related Pathways and Senescence in iPSC-Derived Neuronal Models
Source: bioRxiv. 2025 Jun 15:2025.06.11.659008. Preprint. [Version 1] doi: 10.1101/2025.06.11.659008 (PMC12259118; doi:10.1101/2025.06.11.659008)
Supplement: Supplement 2 — Figure B [file media-2.pdf]

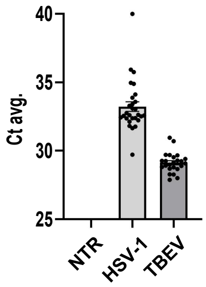

Figure B: Confirmation of viral infection in COs.

Figure B: Confirmation of viral infection in COs. qRT-PCR validation of HSV-1 and TBEV infection in COs harvested at D60. Ct average (CT avg.) is plotted due to non-detectable expression in NTRs. Each dot represents one biological replicate, n≥3. See **Table B** for reference on specific number of samples, replicates and cell line details.
