# Supplementary material for: Viral Infection Induces Alzheimer’s Disease-Related Pathways and Senescence in iPSC-Derived Neuronal Models
Source: bioRxiv. 2025 Jun 15:2025.06.11.659008. Preprint. [Version 1] doi: 10.1101/2025.06.11.659008 (PMC12259118; doi:10.1101/2025.06.11.659008)
Supplement: Supplement 3 — Figure C [file media-3.pdf]

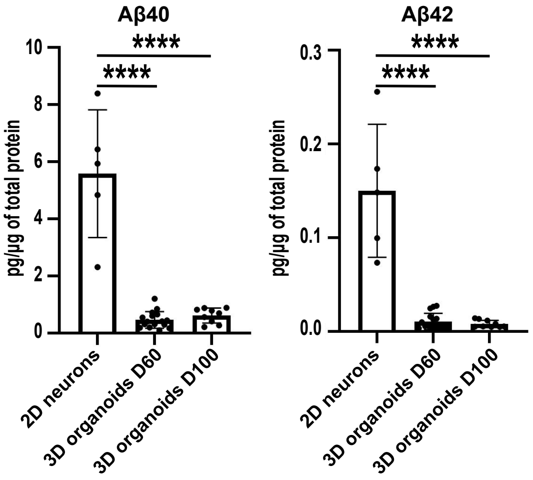

Figure C: Comparison of secreted Aβ peptide levels in 2D neuronal cultures and 3D COs.

Figure C: Comparison of secreted Aβ peptide levels in 2D neuronal cultures and 3D COs. Quantifying Aβ40 and Aβ42 levels in the culture medium of 2D neuronal cultures and 3D COs measured by ELISA. Data represent mean ± SEM from $n \geq 4$ of independent experiments. Statistical analysis was performed using unpaired t-test; ****$p < 0.001$. See **Table B** for reference on specific number of samples, replicates and cell line details.
