# Supplementary material for: Viral Infection Induces Alzheimer’s Disease-Related Pathways and Senescence in iPSC-Derived Neuronal Models
Source: bioRxiv. 2025 Jun 15:2025.06.11.659008. Preprint. [Version 1] doi: 10.1101/2025.06.11.659008 (PMC12259118; doi:10.1101/2025.06.11.659008)
Supplement: Supplement 4 — Figure D [file media-4.pdf]

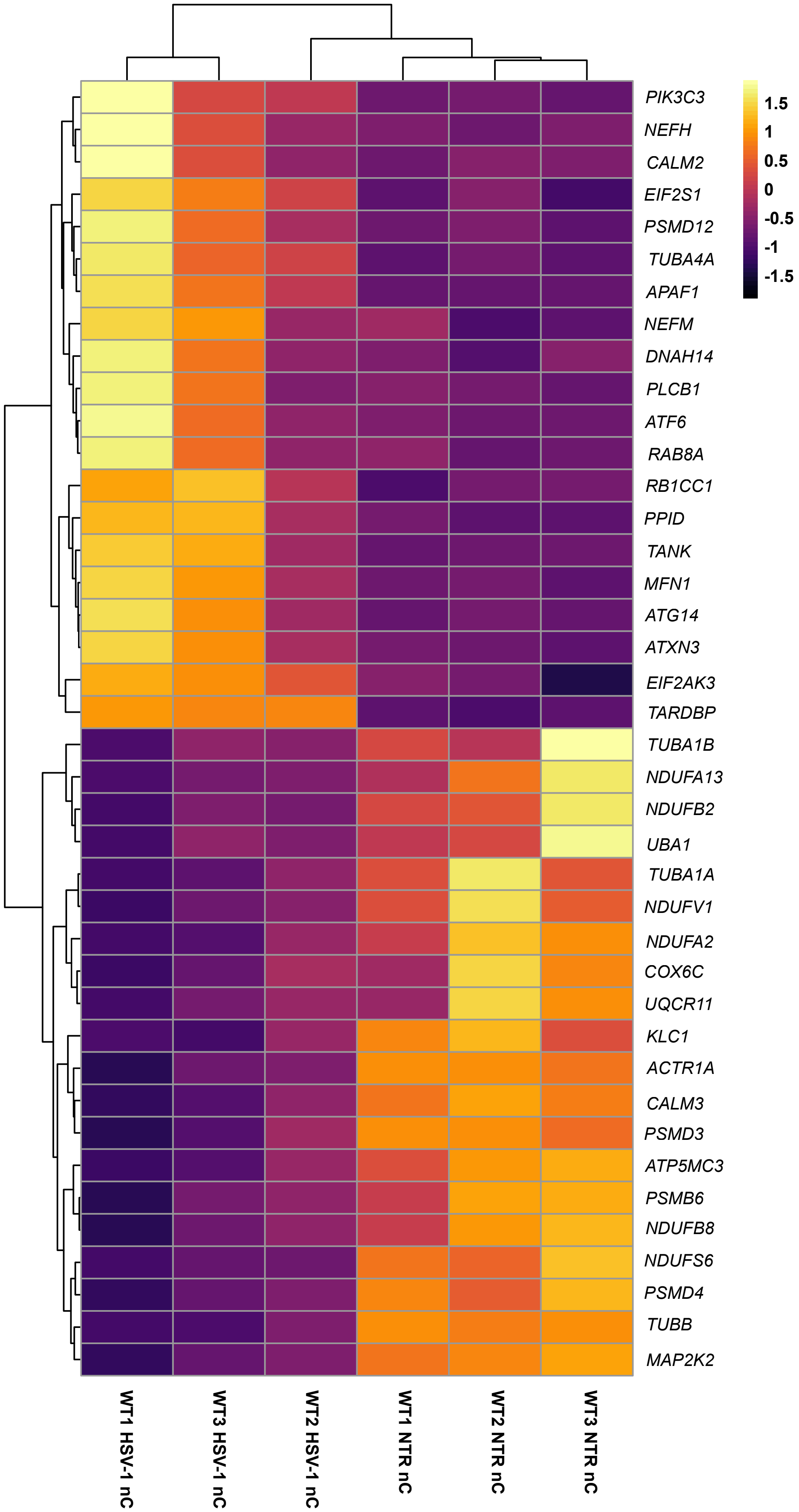


**Figure D: HSV-1-induced transcriptional changes affect genes involved in neurodegeneration-associated pathways.**

**Figure D: HSV-1-induced transcriptional changes affect genes involved in neurodegeneration-associated pathways.** Heatmap displaying the top 20 most upregulated and top 20 most downregulated genes from HSV-1-infected COs when compared to non-infected controls (p.adj<0.5, log2FC>|0.5|, top 40 DEGs selected based on their weighted scores).
