# Supplementary material for: Viral Infection Induces Alzheimer’s Disease-Related Pathways and Senescence in iPSC-Derived Neuronal Models
Source: bioRxiv. 2025 Jun 15:2025.06.11.659008. Preprint. [Version 1] doi: 10.1101/2025.06.11.659008 (PMC12259118; doi:10.1101/2025.06.11.659008)
Supplement: Supplement 5 — Figure E.1, E.2 [file media-5.pdf]

E.1

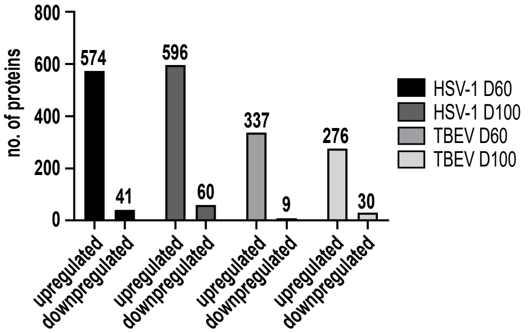

E.2

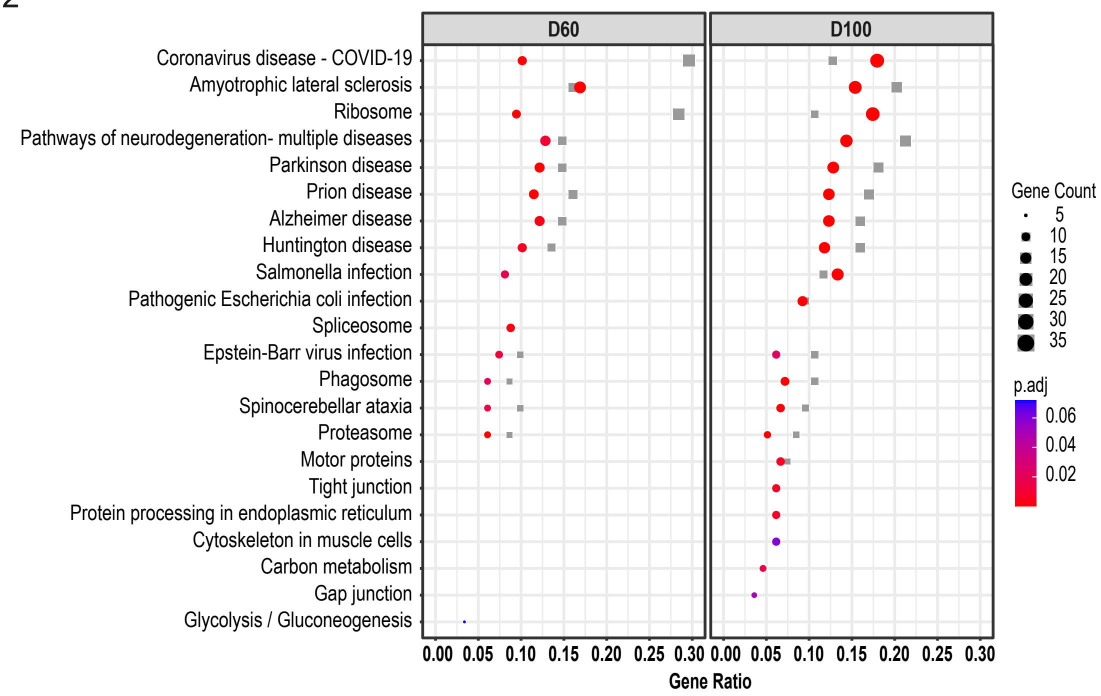

Figure E: Analyses of secretome deregulation in viral-infected COs.

Figure E: Analyses of secretome deregulation in viral-infected COs. (E.1) Bar plot comparing the number of significantly deregulated secreted proteins in D60 and D100 organoids following HSV-1 and TBEV infection. **(E.2)** Top deregulated pathways enriched upon viral infection (HSV-1 and TBEV) based on DSPs related to SASP (HSV-1 infection is shown in color, TBEV in grey scale).
