# Supplementary material for: Viral Infection Induces Alzheimer’s Disease-Related Pathways and Senescence in iPSC-Derived Neuronal Models
Source: bioRxiv. 2025 Jun 15:2025.06.11.659008. Preprint. [Version 1] doi: 10.1101/2025.06.11.659008 (PMC12259118; doi:10.1101/2025.06.11.659008)
Supplement: Supplement 7 — Figure G.1, G.2 [file media-7.pdf]

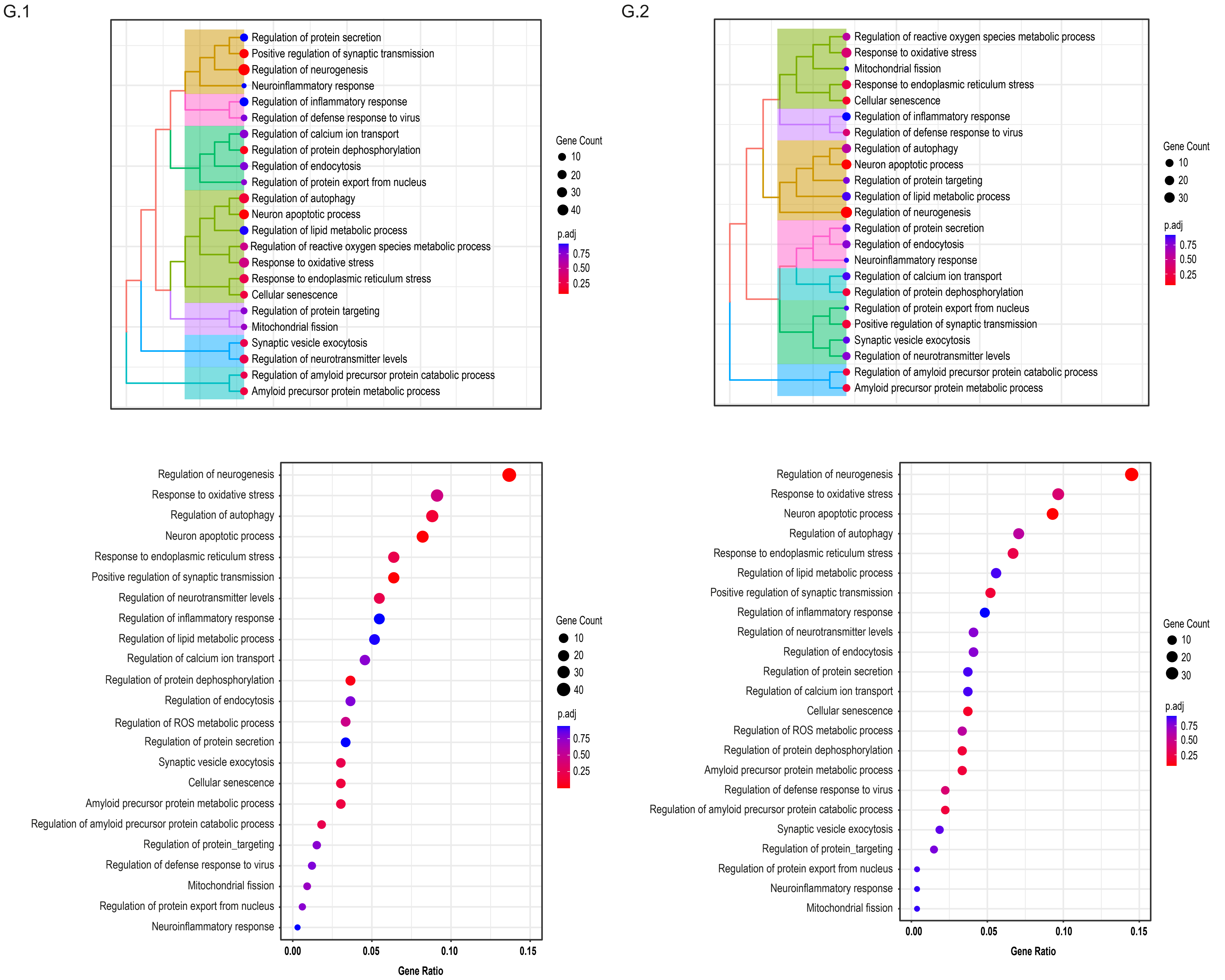


**Figure G: Targeted pathway analysis of HSV-1-infected COs from Rybak-Wolf et al.**

**Figure G: Targeted pathway analysis of HSV-1-infected COs from Rybak-Wolf *et al.*** [42] **(G.1)** Targeted analysis of DEGs in between HSV-1-infected COs and their corresponding non-infected controls with emphasis on literature-defined AD-associated mechanisms ([40–43]; **Table E**) showing strong enrichment in oxidative stress, ER stress, autophagy, apoptosis, synaptic transmission, exocytosis, and senescence—closely matching our analyses in WT and *PSEN1/2* mutant organoids (**Figures 4D and F.4**). **(G.2)** Comparison of targeted AD-related pathway enrichment in acyclovir-treated and untreated conditions. The top charts show clustered data, and the bottom charts show the same data sorted based on p.adj and gene ratio.
